# Listening effort and stress in tinnitus: a multidimensional approach

**DOI:** 10.3389/fnins.2025.1591622

**Published:** 2025-07-07

**Authors:** Giovanna Giliberto, Maria Itati Palacio, Giulia Cartocci, Emiliano Fernandez-Villalba, Dario Rossi, Nieves Minguez, Maria Botia, Jose Domingo Cubillana, Jose Joaquin Ceron, Fabio Babiloni, Maria Trinidad Herrero

**Affiliations:** ^1^Clinical and Experimental Neuroscience (NiCE), Biomedical Research Institute of Murcia (IMIB-Pascual Parrilla), Institute for Aging Research, UniWell, Campus Mare Nostrum, School of Medicine, University of Murcia, Murcia, Spain; ^2^Department of Molecular Medicine, University of Rome “Sapienza”, Rome, Italy; ^3^Department of Otorhinolaryngologist, Virgen of Arrixaca University Clinical Hospital, Murcia, Spain; ^4^Interdisciplinary Laboratory of Clinical Analysis (Interlab-UMU), University of Murcia, Murcia, Spain

**Keywords:** tinnitus, auditory perception, gender differences, EEG analysis, stress biomarkers

## Abstract

**Introduction:**

This study investigated the impact of chronic tinnitus on auditory perception, text comprehension, and physiological stress responses, with a focus on sex-related differences. The main objectives were to assess the influence of sex and stress on tinnitus severity, examine neurophysiological indicators of listening effort, and evaluate the effects of background noise on perceived difficulty and listening pleasantness.

**Materials and methods:**

Forty-seven participants (24 with tinnitus, 23 controls) performed a listening task involving audiobook excerpts presented at different signal-to-noise ratios. Subjective ratings, comprehension scores, and physiological data were collected, including salivary alpha-amylase, electrodermal activity, heart rate, and EEG-based measures of listening pleasantness.

**Results:**

Control participants outperformed tinnitus participants during the initial quiet condition (*p* = 0.020), with male controls scoring significantly higher than males with tinnitus (*p* = 0.008). Tinnitus participants rated listening as less pleasant in both quiet (*p* = 0.036) and high-noise conditions (*p* = 0.012). Female participants reported greater difficulty under moderate noise (*p* = 0.030), while EEG data showed higher enjoyment in males (*p* = 0.005). Salivary amylase increased post-task (*p* = 0.016), electrodermal activity differed between the initial and final quiet phases (*p* < 0.001), and heart rate varied according to noise levels (*p* = 0.008). Negative correlation emerged between subjective and EEG-based pleasantness in the quiet condition.

**Discussion:**

These findings suggest that tinnitus imposes a measurable cognitive and emotional burden, influenced by both sex and stress responses. They emphasize the need for multimodal, personalized, and gender-sensitive approaches in the assessment and management of tinnitus.

## 1 Introduction

Following up on previous research that investigated listening effort in individuals with tinnitus through electroencephalography (EEG) and skin conductance measurements during continuous speech stimuli under varying signal-to-noise ratio (SNR) conditions (Cartocci et al., 2023), the present study aims to further explore the neurophysiological, psychological, and biochemical dimensions of tinnitus. Specifically, it integrates the analysis of stress-related biomarkers and examines gender differences to provide a more comprehensive understanding of the mechanisms underlying tinnitus perception and its impact on quality of life.

It is essential to differentiate between non-distressing and distressing tinnitus. The former is defined as “the conscious perception of a tonal or composite sound in the absence of an identifiable external source,” while the latter involves significant emotional or psychological distress (De Ridder et al., 2021). Two primary types of tinnitus have been identified: subjective tinnitus, a phantom sound perceived without external stimuli, and objective tinnitus, a sound generated within the body that reaches the ear via body tissues (Han et al., 2024; Simoes et al., 2021). Its etiology remains unclear and may be either primary or secondary. Severe forms, often associated with hearing loss, can significantly impair the psychophysical well-being of affected individuals (Jarach et al., 2022; Vasilkov et al., 2023).

Gender and stress are critical modulators of tinnitus. Womens often report greater tinnitus-related distress, potentially due to hormonal modulation of auditory processing and sex-specific coping strategies (Cederroth and Schlee, 2022). Chronic stress has been implicated in both the onset and exacerbation of tinnitus, potentially through dysregulation of the hypothalamic–pituitary–adrenal (HPA) axis (Simões et al., 2021). This may lead to maladaptive neuroplasticity, contributing to symptom severity and impaired habituation (Vanneste and De Ridder, 2016). Stress-related physiological responses involve the sympathetic nervous system and the HPA axis, which can be assessed through salivary alpha-amylase and cortisol levels (Lombardo et al., 2023; Patil et al., 2023; Hillebrand et al., 2024). Listening effort, defined as “the intentional allocation of cognitive resources to overcome auditory obstacles during task execution” (Pichora-Fuller, 2016), is crucial for understanding communication difficulties in individuals with tinnitus, hearing loss, or age-related auditory decline (Francis and Love, 2020). The Speech-in-Noise paradigm is commonly used to evaluate the cognitive demands associated with distinguishing speech from background noise (Quaranta et al., 2018). EEG and electrodermal activity (EDA) are employed to quantify listening effort. EEG studies highlight alpha band modulations in centro-parietal and occipito-parietal regions as indicators of auditory cognitive load (Cartocci et al., 2018; Halgren et al., 2019; Arioli et al., 2024). Reduced parietal alpha power is linked to greater effort during speech-in-noise tasks, while increased posterior alpha activity is observed in individuals reporting heightened listening difficulty (Paul et al., 2021; Cartocci et al., 2023; Zhang et al., 2023; Klatt et al., 2022). EDA, reflecting autonomic nervous system activity, complements EEG by revealing increased skin conductance under higher cognitive load, particularly in individuals with hearing loss (Francis et al., 2021; Bsharat-Maalouf et al., 2023). To investigate these mechanisms in an ecologically valid context, normal-hearing participants with tinnitus were exposed to continuous auditory stimuli both in silence and under varying background noise levels using an audiobook to simulate real-life scenarios. Measures included EEG, EDA, a visual analog scale (VAS) for perceived difficulty and pleasantness, and standardized questionnaires: the Tinnitus Handicap Inventory (THI) (Cuesta et al., 2022), the Tinnitus Questionnaire 12 (TQ12) (Manchaiah et al., 2020), and a hyperacusis questionnaire (Herráiz et al., 2006), acknowledging the frequent co-occurrence of sound hypersensitivity (Cederroth and Schlee, 2022). The State–Trait Anxiety Inventory (STAI) was also administered to assess anxiety levels in comparison to a healthy control group (Capdevila-Gaudens et al., 2021). The primary aims of this study were to examine how sex and gender-related factors and stress influence tinnitus incidence and severity, to evaluate neurophysiological markers of listening effort in individuals with tinnitus versus controls, and to assess the impact of background noise on subjective auditory experience. This integrative framework offers a refined perspective on the cognitive and psychological burden of tinnitus and its influence on auditory processing.

## 2 Materials and methods

### 2.1 Participants

This investigation enrolled a total of 47 participants, comprising two distinct groups: 24 patients diagnosed with chronic tinnitus (TIN group) and 23 normal hearing control participants (CTRL group). The TIN cohort consisted of 14 women and 10 men, with a mean age (± standard deviation) of 54.75 ± 15.16 years. The CTRL group included 14 women and 9 men, with a mean age of 60.17 ± 13.75 years.

Inclusion criteria for all participants encompassed normal auditory function; absence of significant psychiatric or neurological disorders, or any anatomo-functional alterations that could potentially confound the study results; and no current use of psychoactive medications, explicitly defined as no consumption of such substances for a minimum period of 3 months prior to participation. For the TIN group, an additional criterion stipulated the perception of tinnitus as the primary symptom (either unilateral or bilateral) for a minimum duration of 3 months.

### 2.2 Self-report questionnaires and psychometric assessments

Individuals diagnosed with tinnitus were assessed using two psychometric questionnaires to evaluate the impact of tinnitus on quality of life and the severity of tinnitus-related distress, two psychometric questionnaires were employed: the Tinnitus Handicap Inventory (THI) (Soriano-Reixach et al., 2023) and the Tinnitus Questionnaire (TQ12) (Soriano-Reixach et al., 2023). The THI, a 25-item psychometric tool, evaluates the impact of tinnitus on quality of life across three main domains (functional, emotional, and catastrophic). Based on the score, the distress is classified into increasing severity levels. Responses are scored as Yes (4), Sometimes (2), or No (0), yielding a total score ranging from 0 to 100. The Spanish version demonstrates high internal consistency (Cronbach’s *α* ≈ 0.93) and good test–retest reliability. It is widely used in clinical settings to assess the impact of tinnitus on daily life (Newman et al., 1996; Cuesta et al., 2022).

The TQ12, a 12-item psychometric tool derived from the full version of the Tinnitus Questionnaire, measures tinnitus’s cognitive and emotional impact. It is validated for sensitivity to change and is suitable for clinical and research purposes, making it useful for monitoring therapeutic outcomes and classifying tinnitus-related distress. The total score ranges from 0 to 24, with higher scores indicating greater discomfort associated with tinnitus (Manchaiah et al., 2020; Simões et al., 2021). The Spanish adaptation maintains good internal consistency (Cronbach’s *α* ≈ 0.85–0.90) and is validated for use in Spanish-speaking populations.

To quantify abnormal sound sensitivity and evaluate the cognitive, emotional, and behavioral impact of tinnitus, the hyperacusis questionnaire was utilized. It comprises 14 items, each rated on a 4-point Likert scale, allowing for a nuanced evaluation of the individual’s experience. A total score exceeding 28 is generally considered indicative of clinically relevant hyperacusis. The Spanish translation of the Geräuschüberempfindlichkeit (G-ÜF), has been validated for clinical monitoring, demonstrating high reliability and internal consistency (Herráiz et al., 2006).

The State–Trait Anxiety Inventory (STAI), a 40-item questionnaire, was employed to evaluate the two dimensions of anxiety: state anxiety (a temporary emotional condition) and trait anxiety (a stable predisposition to experience anxiety) (Knowles and Olatunji, 2020; Capdevila-Gaudens et al., 2021). Each item is rated on a 4-point Likert scale, with total scores ranging from 20 to 80 for each subscale. The Spanish version exhibits good internal consistency (Cronbach’s *α* ≈ 0.89) and construct validity, making it suitable for assessing hypersensitivity to sound in Spanish-speaking individuals.

To further assess participants’ subjective experiences during the auditory task, a Visual Analogue Scale (VAS) was employed to measure perceived difficulty and pleasantness of the auditory stimuli at different stages of the audiobook listening session. The VAS consisted of a 10 cm horizontal line, with endpoints labeled “no difficulty”/“not at all pleasant” on the left and “maximum difficulty”/ “extremely pleasant” on the right. Participants were instructed to indicate a point along the line that best reflected their experience, yielding a continuous measure of their cognitive and emotional responses.

Importantly, it was clarified to participants that the VAS ratings for both pleasantness and difficulty referred specifically to the content of the audio passages presented during the listening task. For each condition, they were asked to rate how pleasant or how difficult they found the listening experience.

The VAS was administered in paper format, and responses were recorded manually. The position of each mark was measured with a standard ruler, yielding values on a 0–100 scale with a precision of ±1 mm. This method, widely used in psychophysical and clinical research, provides high sensitivity to subtle perceptual changes and allows for quick and reliable assessments of subjective experience (Wu et al., 2023).

### 2.3 Experimental protocol

The experimental protocol, based on a previously conducted pilot study (Cartocci et al., 2023), involved listening to an audiobook while simultaneously recording electroencephalographic (EEG) and autonomic data. The auditory stimulus, “*El ingenioso hidalgo Don Quijote de La Mancha, Parte 1 - Capitulo 1*,” was sourced from the “AlbaLearning” database. The total duration of the auditory stimulation was 11 min and 40 s, including three signal-to-noise ratio (SNR) conditions (0, +5, and +10 dB) presented in a randomized order among participants, with an average duration of 1 min and 30 s per condition. Quiet conditions were included at the beginning and at the end of the audiobook, each lasting approximately 2 min and 30 s.

The SNR-modified audio tracks were prepared using Audacity software (Audacity, 2025). The background noise consisted of “babble noise”(Turrini et al., 1993), a widely used auditory stimulus in neuroscientific studies involving participants with normal hearing and hearing impairments (Cartocci et al., 2024; Cartocci et al., 2021). Auditory stimulation was delivered at 65 dB SPL by two loudspeakers positioned 1 m in front of each participant, aligned at face level (Cartocci et al., 2021).

**Figure 1:**
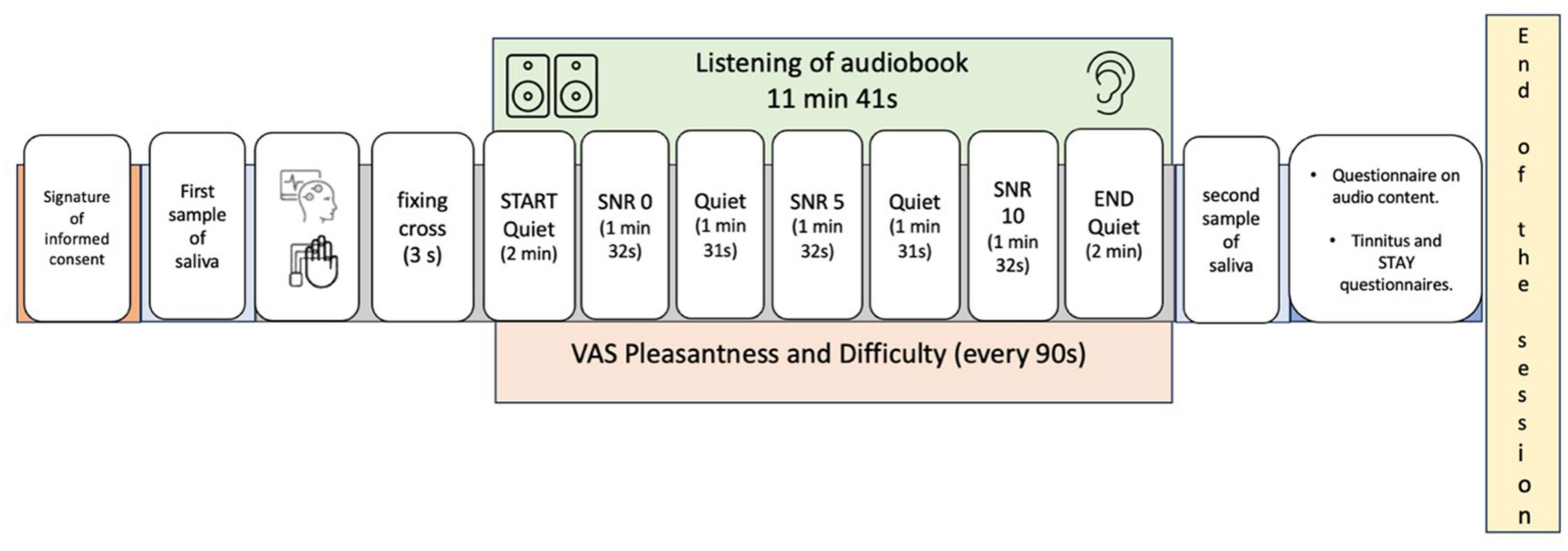
Graphical representation of the experimental protocol adopted in the present study. Auditory conditions during audiobook listening were assigned to participants in a pseudorandomized manner, following the sequence Quiet–signal-to-noise ratio (SNR)–Quiet–SNR–Quiet–SNR–Quiet, for a total of seven conditions. Every 90 s, participants completed two visual analog scales (VAS): one for perceived pleasantness and the other for perceived difficulty. Additionally, at the end of the audiobook, questions were administered to assess text comprehension.

Prior to auditory stimulation, participants fixed on a white screen for 3 s (pre-stimulus phase). At regular 90-s intervals, corresponding to the seven conditions (quiet and three SNR levels), participants rated the pleasantness and perceived difficulty of the auditory task using two distinct Visual Analogue Scales (VAS) ranging from 0 to 100. These scales have been previously validated for use with tinnitus patients (Zhang et al., 2023). Following the auditory stimulation, participants completed a multiple-choice questionnaire assessing their comprehension of the audiobook content (Supplementary Table 1), providing behavioral data on task engagement and performance (Figure 1).

### 2.4 Saliva analysis

Prior to and following the audiobook listening session, participants provided saliva samples to assess potential changes in biochemical markers associated with stress and anxiety. The analysis specifically focused on measuring salivary amylase and cortisol levels, which are well-established indicators of physiological stress responses. This procedure aimed to evaluate the potential impact of the auditory stimulus on participants’ stress and anxiety levels by comparing biomarker concentrations before and after exposure (Hillebrand et al., 2024). All saliva samples were collected between 8:30 and 11:00 a.m. to control for diurnal variation in biomarker levels.

The collection and analysis of amylase and cortisol in saliva involves a meticulous and standardized process (Chojnowska et al., 2021). Saliva samples are typically obtained through the passive drool method, avoiding contamination, and following specific guidelines such as refraining from eating, drinking, or brushing teeth for at least 30–60 min before collection. Once collected, the samples were rapidly frozen at −80°C to preserve their integrity. For analysis, the samples were thawed, centrifuged, and the supernatant was used for the measurement of analytes. An automated spectrophotometric assay was used for the alpha-amylase measurements whereas a chemiluminescence assay was used for cortisol. Both assays have been previously validated in human saliva (Sabater-Gárriz et al., 2024). The results are interpreted by comparing them with established reference ranges, considering factors such as the time of the day and clinical context (Xie et al., 2022). This non-invasive approach provides valuable insights into the functioning of the hypothalamic–pituitary–adrenal axis and the sympathetic nervous system.

### 2.5 EEG signal acquisition and processing

Electroencephalographic (EEG) signals were acquired using the Mindtooth Touch headset, equipped with eight water-based electrodes (saltwater sponges and passive Ag/AgCl electrodes) (Sciaraffa et al., 2022), placed, according to the 10–20 system (Furlong et al., 2021) in prefrontal (AFz, AF3, AF4, AF7, AF8) and parietal (Pz, P3, P4) cortices plus ground (left mastoid) and reference (right mastoid). This device has been previously employed for EEG recording in the analysis of psychophysiological variables in neuroscientific studies.

The acquired EEG signal at a sampling frequency of 125 Hz, underwent initial filtering using a fifth-order Butterworth filter with a passband of 2–30 Hz. Blink artifacts were identified and removed using the Reblinca method (Jiang et al., 2019). From the artifact-free EEG data, the Global Field Power (GFP) was calculated, representing the spatial standard deviation, and quantifying the overall activity at each time point by simultaneously considering data from all recording electrodes. The analysis focused on the Alpha frequency band (8–13 Hz), defined based on the Individual Alpha Frequency (IAF) (Sattelberg J. et al., 2024). For each participant, the frequency range of interest was tailored to (IAF-4, IAF + 2), determined from a one-minute recording with eyes closed prior to the start of the experiment.

### 2.6 Acquisition and processing of autonomic activity signals

Electrodermal activity (EDA) was recorded using the NeXus-10 biofeedback system (Mind Media BV, Netherlands), connected via Bluetooth to a laptop running the proprietary BioTrace+ software. EDA measurements were obtained using a NeXus sensor equipped with two Ag/AgCl electrodes embedded in Velcro straps, which were securely attached to the palmar surfaces of the index and middle fingers of the participant’s non-dominant hand (Boucsein et al., 2012). The analysis of the tonic component of skin conductance (Skin Conductance Level, SCL) was conducted directly using the BioTrace software integrated with the Nexus system. The SCL represents the slowly varying baseline of the EDA signal and reflects gradual changes in autonomic arousal over time (Mukhopadhyay et al., 2022).

### 2.7 Statistical analysis

Non-parametric methods were employed for the statistical analysis of this study. Friedman’s ANOVA was used for within-group comparisons, while Mann–Whitney U tests were applied for between-group comparisons. Spearman’s Rank Order correlations were conducted to examine the relationships between neurophysiological values, questionnaire scores, and evaluations. In the pre-stimulation phase, the mean neurophysiological activity during the 3 s preceding the listening phase was used. For the quiet condition, the average of the four quiet periods in the experimental protocol was calculated.

## 3 Results

The results of this study can be categorized into subjective and objective evaluations. Subjective assessments encompassed the text comprehension questionnaire, perceived pleasantness, and difficulty scores (VAS), and self-reported questionnaires (THI, mini-TQ, HI, and STAI). Objective parameters included values obtained from EEG analysis (Pleasantness, Alpha P3, Alpha P4, Beta P3P4, and mental engagement), electrodermal values (GSR and HR), and salivary biomarkers (amylase and cortisol) (Table 1).

**Table 1:** Summary of key findings across subjective (VAS ratings, questionnaires) and objective (EEG, salivary biomarkers, EDA, HR) measures, with group, gender, and SNR-related effects highlighted where significant.

Category	Topic	Key findings
Subjective evaluations	Correct answers to the comprehension questionnaire	Lower comprehension scores in tinnitus men under noisy conditions (SNR5, SNR10).
	Pleasantness perceived through the analogue visual scale	Pleasantness (quiet): Lower pleasantness in tinnitus group, especially in men. Pleasantness (SNR0): Lowest scores in TIN men; highest in CTRL men.
	Difficulty perceived through the analogue visual scale	Difficulty (SNR0/SNR5): Higher perceived difficulty in women, especially in TIN group. Difficulty (SNR10/quiet): No significant differences; trend in silence phase.
	Self-reported questionnaire	No group or gender differences (THI, mini-TQ, HI, STAI).
Objective evaluations	EEG analysis	EEG (pleasantness): Higher EEG pleasantness in men, independent of group. EEG (other metrics): No significant effects; mental engagement near significance.
	Salivary biomarkers	Amylase: Significant post-test increase, independent of group/sex. Cortisol: No significant changes observed.
	Electrodermal activity	EDA (quiet): Significant reduction from initial to final silent phase. EDA (SNR): Significant differences across SNR levels; not in silent phases.
	Heart rate	Significant differences across SNR and silence.
Correlation analysis	Statistical correlations	VAS–EEG (pleasantness): Negative correlation in silent phase. Amylase–SNR: Positive correlation with SNR5 and SNR10. STAI–HR: Negative correlation between trait anxiety and HR in SNR10.

### 3.1 Subjective evaluations

#### 3.1.1 Correct answers to the comprehension questionnaire

Analysis of subjective results utilized the Mann–Whitney U test to compare the number of correct responses across different experimental conditions (Quiet, SNR10, SNR5, and SNR0). Statistically significant differences were observed when comparing the initial quiet phase of the audio. Specifically, the number of correct responses during the initial quiet phase of the auditory task, stratified by group (TIN and CTRL) and gender (men (M) and women (W)). A statistically significant difference was observed between the TIN and CTRL groups (*p* = 0.020*), with control participants achieving a higher number of correct responses compared to tinnitus patients. Additionally, a significant interaction between gender and group was identified (*p* = 0.008**). The men participants in the control group (CTRL-M) demonstrated a significantly greater number of correct responses compared to men participants in the tinnitus group (TIN-M). No significant differences were observed between women participants in the two groups (TIN-W vs. CTRL-W). These findings suggest that tinnitus may differentially affect auditory comprehension in men during the initial quiet phase of auditory tasks. This pattern was not observed in the other quiet phases along the audio.

During the experimental conditions with background noise, the analysis of correct response values revealed statistically significant differences in the SNR10 (*p* = 0.036*) and SNR5 (*p* = 0.033*) condition. The two groups compared were individuals with tinnitus (TIN) and control participants (CTRL).

In both conditions, control participants (CTRL) exhibited significantly higher correct response rates compared to those with tinnitus (TIN). The control group achieved a mean correct response score slightly above 2.0, whereas the tinnitus group had a lower mean, around 1.8. A similar pattern was observed in the SNR5 condition, where the control group again outperformed the tinnitus group, with mean values of approximately 2.0 and 1.5, respectively.

The TIN group exhibited lower accuracy in auditory tasks under moderate noise conditions, reinforcing the impact of tinnitus on speech perception in noisy environments.

#### 3.1.2 Pleasantness perceived through the analogue visual scale

Regarding perceived pleasantness, as measured by the Visual Analogue Scale (VAS) across different experimental conditions, a statistically significant difference was identified in the quiet phase in relation to the group variable (*p* = 0.036*). Overall, control participants (CTRL) reported higher perceived pleasantness compared to those with tinnitus (TIN), specifically, within the TIN group, men reported a lower mean pleasantness score (57.46) compared to women (68.46) In the control group, both men and women exhibited higher pleasantness ratings, with men averaging close to 76.33 and women at 76.04. The participants with tinnitus perceive auditory stimuli as less pleasant in quiet conditions compared to control participants, reinforcing the impact of tinnitus on auditory perception and comfort in silent environments.

Within the background noise conditions, a significant group-related difference in perceived pleasantness was found in the SNR0 phase (*p* = 0.012*), specifically, the “TIN” condition demonstrates relatively lower pleasantness ratings for both levels, with “M” exhibiting the lowest score among all groups. Conversely, the “CTRL” condition displays markedly elevated pleasantness scores, with “M” in this condition achieving the highest rating overall. However, further statistical analysis would be needed to determine if the differences between “M” and “W” within each condition are also significant, and to quantify the effect size associated with these observed differences.

#### 3.1.3 Difficulty perceived through the analogue visual scale

Statistically significant differences were found concerning perceived difficulty in the SNR0 and SNR5 phases, specifically in relation to the gender variable (*p* = 0.029* and *p* = 0.030* respectively).

In both noise conditions, a statistically significant difference was found for the gender variable. Specifically, women reported significantly higher perceived difficulty compared to men across both groups. This effect is particularly pronounced within the TIN group, where women participants exhibited the highest difficulty ratings, exceeding 35 in both conditions. In contrast, men participants in the TIN group reported lower perceived difficulty scores, around 20 in SNR0 and slightly above 20 in SNR5. Similarly, in the CTRL group, women participants consistently rated difficulty higher than men, although the overall perceived difficulty was lower than in the TIN group. Men in the control group reported the lowest perceived difficulty, with mean values below 15 in both SNR0 and SNR5 conditions.

In these phases, women participants reported higher perceived difficulty compared to men participants. However, no significant differences were found for the group variable in these phases. Furthermore, in the SNR10 and quiet conditions, no statistically significant differences were observed, although the quiet condition approached significance (*p* = 0.056), suggesting a potential trend that warrants further investigation.

#### 3.1.4 Self-reported questionnaire

Finally, no statistically significant differences were detected among participants concerning the THI, mini-TQ, HI, and STAI questionnaire scores, indicating that these self-reported measures did not exhibit group- or gender-related variations.

### 3.2 Objective evaluations

#### 3.2.1 EEG analysis

**Figure 2:**
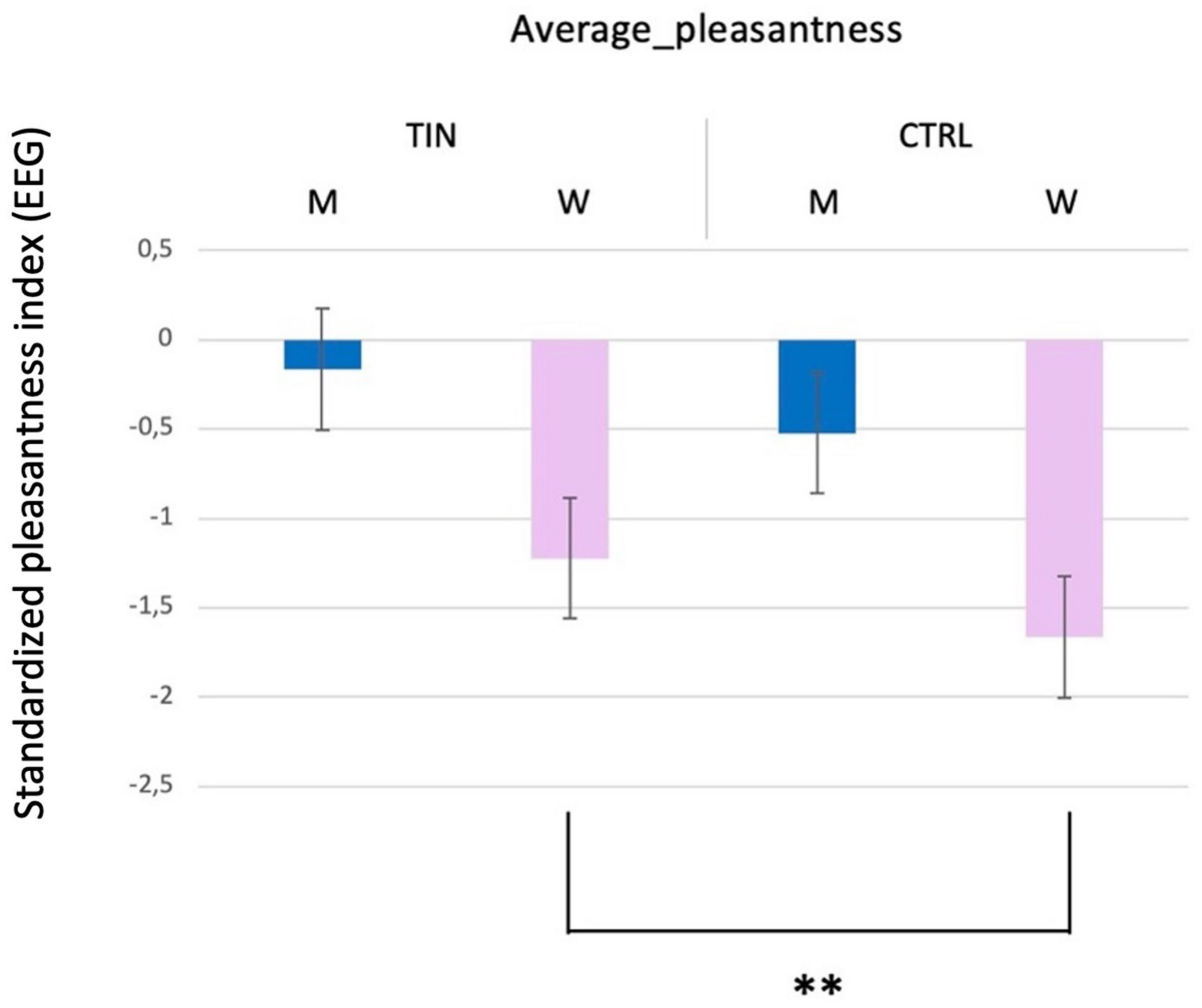
Average pleasantness ratings across experimental conditions. Bar graph shows mean pleasantness scores (± SEM) for man (M) and woman (H) TIN and CTRL participants.

Secondly, concerning the objective results obtained through EEG analysis, a statistically significant difference was identified in perceived pleasantness (*p* = 0.005**; Mean men with tinnitus = −0.165 and SD = 1.167; Mean women with tinnitus = −1.224 and SD = 1.528; Mean control men = −0.524 and SD = 0.775; Mean control women = −1.663 and SD = 1.206) (Figure 2), Perceived pleasantness refers to an EEG-derived metric provided by the recording device. Men participants exhibited higher levels of EEG-based pleasantness compared to men participants. However, no statistically significant differences were found concerning the group variable. Figure 2 illustrates this effect, with men showing consistently higher pleasantness scores than women across the conditions tested.

For other electroencephalographic parameters analyzed, including Alpha P3, Alpha P4, Beta P3 P4, and mental engagement, no statistically significant differences were observed. No statistically significant differences were observed in the mental engagement parameter across groups.

#### 3.2.2 Salivary biomarkers

**Figure 3:**
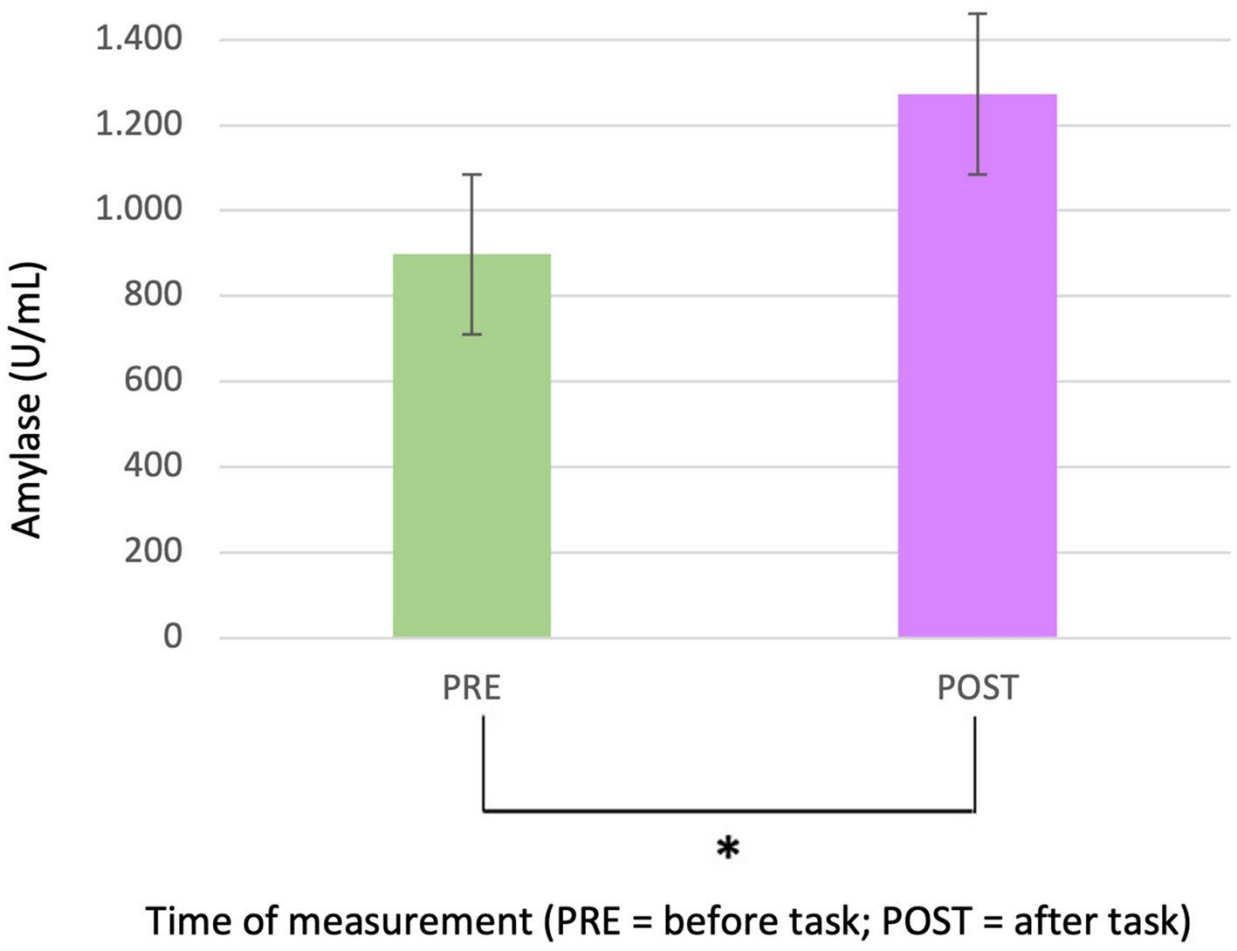
Bar graph referring to pre and post hearing amylases.

Salivary analysis revealed a statistically significant increase in amylase levels post-test (*p* = 0.016*; Mean amylase levels pre-test = 898 and SD = 695; Mean amylase levels post-test = 1.273 and SD = 975) (Figure 3), indicating an overall rise in amylase concentration after the auditory stimulus. This effect was independent of both sex and group variables. Figure 3 presents a visual representation of these findings, highlighting the increase in amylase levels from pre- to post-treatment. In contrast, no statistically significant differences were detected in cortisol levels.

#### 3.2.3 Electrodermal activity

**Figure 4:**
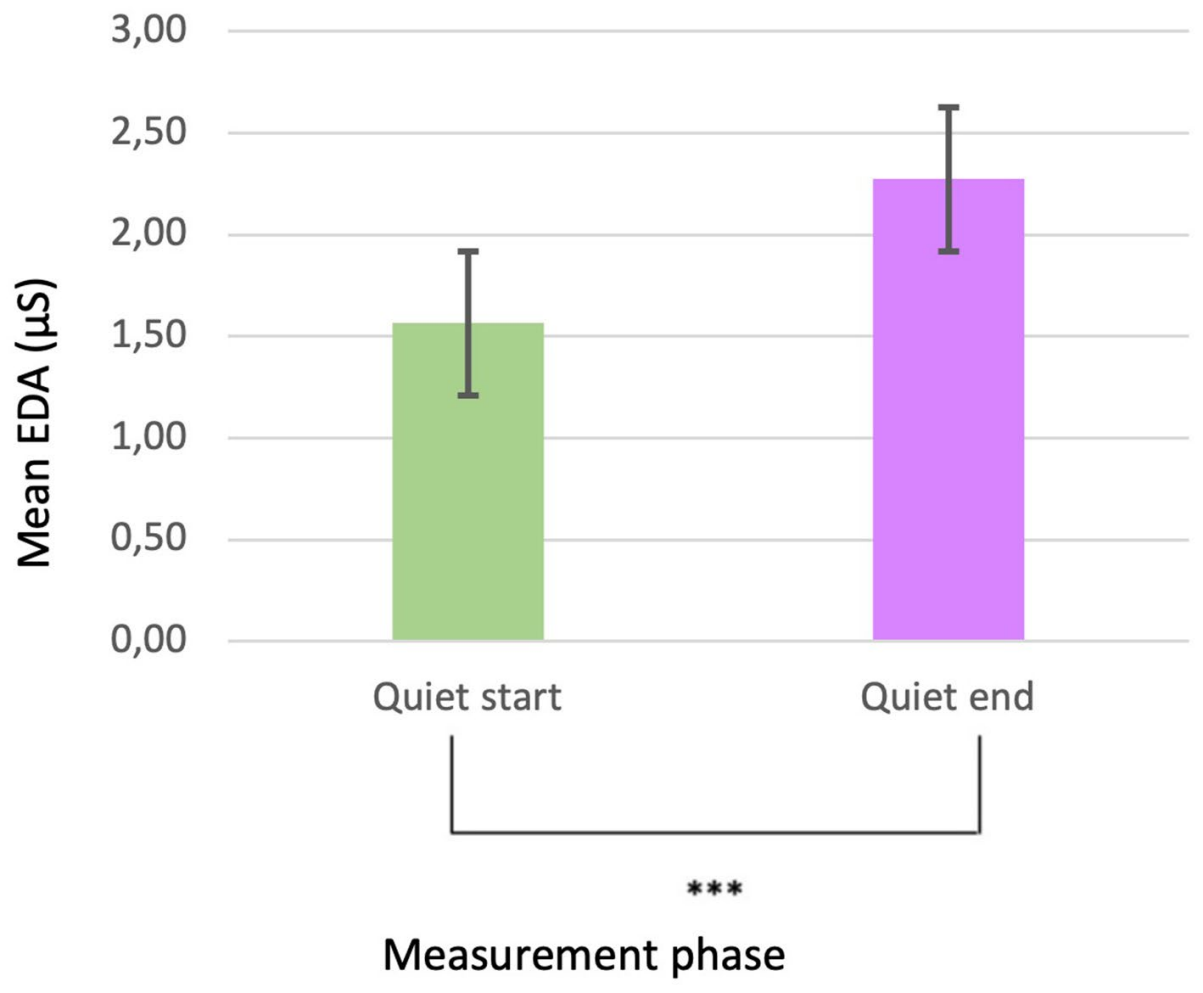
Bar graph comparing EDA values of the quiet at start and end of listening.

The analysis of EDA demonstrated a significant difference (*p* < 0.001***; Mean EDA start level = 1,566 and SD = 1.569; Mean EDA end levels = 2,275 and SD = 1.558) (Figure 4) between the initial quiet phase and the final phase, irrespective of sex and group. Figure 4 illustrates the notable reduction in electrodermal activity, indicating a physiological adaptation or habituation effect over time.

**Figure 5:**
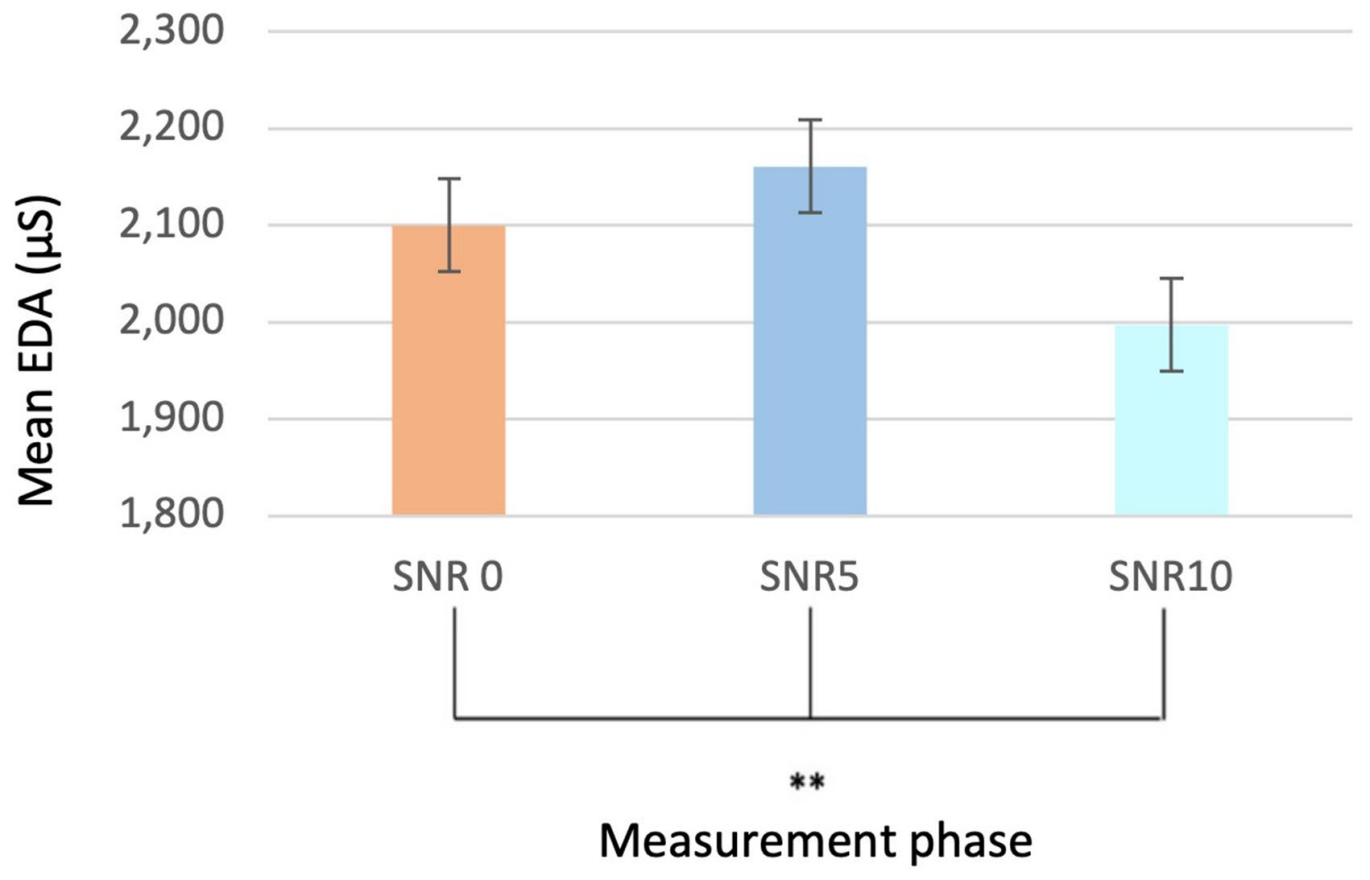
Bar graph comparing EDA values during different noise phases (SNR0, SNR5, SRN10).

Similarly, within the different SNR phases (SNR0, SNR5, and SNR10), statistically significant differences were observed, independent of sex and group (*p* < 0.01**; Mean EDA SNR0 2,100 and SD = 1.476; Mean EDA SNR5 2,161 and SD = 1.543; Mean EDA SNR10 = 1,997 and SD = 1.502) (Figure 5). However, no significant differences were found in the quiet phases during the auditory stimulus.

#### 3.2.4 Heart rate

**Figure 6:**
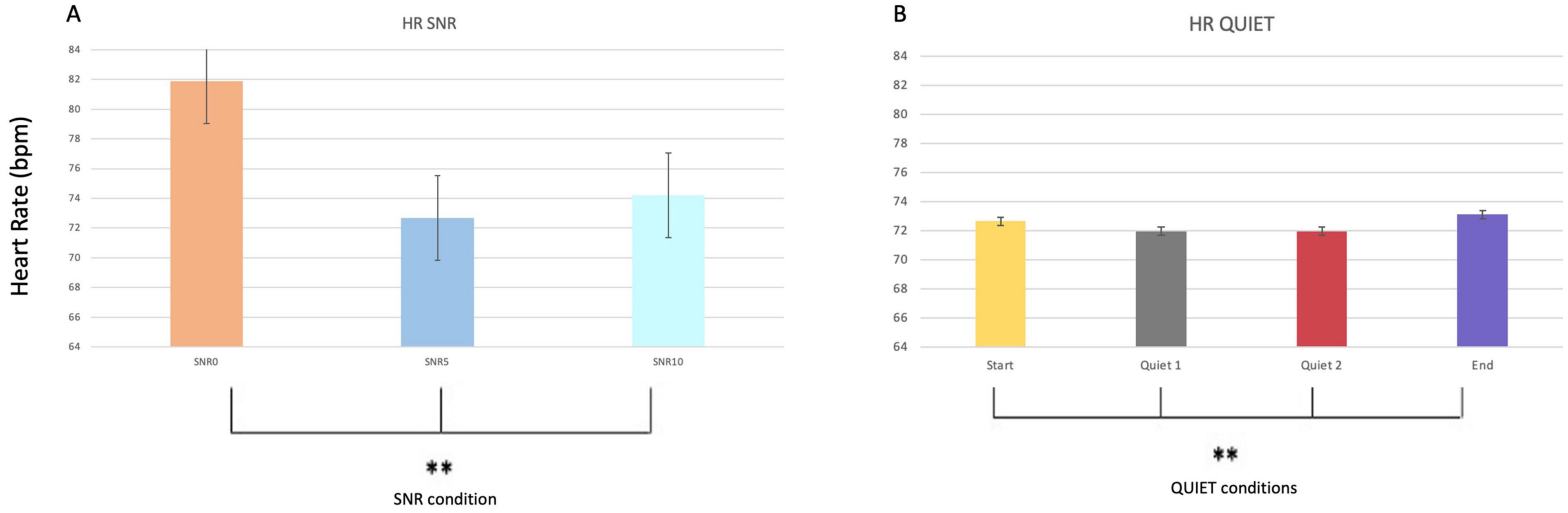
Bar graphs comparing HR values during the different noise phases **(A)** (SNR0, SNR5, SRN10) as well as during the quiet phases **(B)**.

Regarding cardiac rhythm parameters, statistically significant effects were found across different SNR conditions (*p* = 0.008**; Mean SNR0 = 81.8 and SD = 20.011; Mean SNR5 = 72.6 and SD = 11.255; Mean SNR10 = 74.2 and SD = 11.170), regardless of sex and group. Additionally, no statistically significant effect of sex was observed for this parameter. Statistically significant results were also observed during quiet conditions (*p* = 0.036*; Mean Start = 73 and SD = 11.315; Mean Quiet1 = 72 and SD = 11.760; Mean Quiet 2 = 72 and SD = 11.760; Mean End = 73 and SD = 11.533) (Figures 6A,B). Figures 6A,B depict these variations, demonstrating distinct changes in cardiac activity in response to varying SNR levels and during quiet phases.

### 3.3 Correlation analysis

A negative correlation was identified between perceived pleasantness during quiet conditions and the average EEG-recorded pleasantness levels (*p* = 0.040*; Pearson’s *r* = −0.258). Specifically, as subjective pleasantness ratings increased (measured via VAS), the corresponding pleasantness levels (measured with Mindtooth) tended to decrease. This relationship suggests a potential discrepancy between self-reported and neurophysiological measures of pleasantness.

A positive correlation was found between post-auditory salivary amylase levels and the SNR5 (*p* = 0.012*; Pearson’s *r* = 0.377) and SNR10 (*p* = 0.017*; Pearson’s *r* = 0.359) noise conditions, indicating that salivary amylase responses are more strongly associated with the presence of background noise. This suggests that physiological activation, as measured by salivary amylase, may be more influenced by the acoustic environment than by subjective pleasantness in quiet conditions.

Additionally, a negative correlation was found between STAI trait anxiety scores and heart rate (HR) measured during the HR SNR10 phase (*p* = 0.027*; Pearson’s *r* = −0.333). This finding suggests that individuals with higher trait anxiety levels exhibit lower HR during this condition, potentially reflecting a physiological adaptation to stress or heightened autonomic regulation.

## 4 Discussion

The findings of this study provide novel insights into the effects of tinnitus on auditory perception, speech comprehension, and physiological responses under various listening conditions. The observed decline in speech comprehension performance among individuals with tinnitus, particularly in quiet environments and at moderate noise levels (SNR10 and SNR5), suggests a potential disruption in the cognitive processes involved in auditory stimulus processing. This aligns with the neuropsychological model of tinnitus proposed by Tzounopoulos et al. (2019), which posits that tinnitus interferes with central auditory processing. Notably, this impairment was not evident under high-noise conditions (SNR0), implying that excessive environmental noise may mask the impact of tinnitus.

Gender differences in tinnitus perception and difficulty in high-noise environments (SNR0 and SNR5) were significant, with women more frequently reporting heightened discomfort compared to men. These findings may be associated with a higher susceptibility to tinnitus and increased emotional distress, particularly in relation to sleep disturbances and auditory perception (Attanasio et al., 2013; Niemann et al., 2020; Simões et al., 2021), as well as with a lack of habituation in tinnitus patients in response to auditory stimulation (Cartocci et al., 2012).

The general increase in salivary alpha-amylase levels among all participants, regardless of tinnitus status, indicates that noise exposure triggers an acute stress response mediated by sympathetic nervous system activation (Ali and Nater, 2020). This physiological reaction underscores the importance of considering acute stress as a critical factor in the management of auditory disorders, including tinnitus.

The discrepancy observed between subjectively reported pleasantness and that recorded via electroencephalography (EEG), particularly among women, may be interpreted through the lens of gender-specific psychological and social dynamics. This phenomenon could be linked to the concept of gender desirability bias, which suggests that social and cultural expectations influence how individuals, especially women, report subjective experiences (Ng et al., 2024). In the context of tinnitus and noise perception, women may consciously or unconsciously align their responses with perceived social norms, potentially underreporting negative experiences or overstating positive ones.

The correlations identified in this study offer further insights into the complex nature of tinnitus perception. In particular, the negative correlation between perceived pleasantness in silent conditions and EEG-recorded pleasantness levels is especially noteworthy. This divergence between subjective and objective measures highlights the multifaceted nature of tinnitus perception and the need for an integrative assessment approach.

Moreover, the positive correlations between post-auditory salivary amylase levels and perceived pleasantness, both during SNR phases and in silence, with a stronger effect in the latter, suggest that physiological stress, as indicated by increased salivary amylase, may paradoxically be associated with heightened pleasantness. This could indicate a compensatory or adaptive mechanism in individuals exposed to stress-inducing auditory stimuli.

Finally, the negative correlation between State–Trait Anxiety Inventory (STAI) scores and heart rate (HR) across various experimental phases (Start HR, silence 1, silence 2, HR SNR10, and final silence) warrants further investigation. This inverse relationship between trait anxiety and cardiac response may reflect a physiological adaptation mechanism in individuals with elevated anxiety levels or suggest a dissociation between the subjective experience of anxiety and its physiological manifestations in auditory contexts (Correia et al., 2023).

The complex correlations found in this study underscore the importance of employing a multimodal evaluation strategy in tinnitus and auditory perception research, integrating subjective, objective, and psychological measures to provide a holistic understanding of tinnitus-related experiences. They also point to the need for further research, as outlined below.

## 5 Future research directions

### 5.1 Gender-based differences in tinnitus

Neurophysiological studies suggest that women with tinnitus exhibit higher beta activity in the prefrontal cortex, indicating a more emotionally charged response to tinnitus (Simões et al., 2021). Furthermore, gender influences treatment outcomes, with women responding more favorably to high-definition transcranial direct current stimulation (HD-tDCS), whereas men show greater improvement with tinnitus retraining therapy (Niemann et al., 2020).

Additionally, previous research indicates that women with tinnitus often display distinct psychological response profiles compared to men, including higher levels of distress and depression, as well as differences in coping strategies and treatment responses (Niemann et al., 2020; Vesely and Klöckner, 2020). These findings underscore the importance of considering gender as a critical variable when interpreting self-reported data in tinnitus research. Such biases may obscure the true extent of the condition’s impact on women, highlighting the need for objective measures, such as EEG-based indices, to complement subjective assessments. This approach could help disentangle the influence of social desirability from genuine perceptual and emotional experiences.

Our results emphasize the need for future gender-sensitive research in the evaluation and treatment of tinnitus.

### 5.2 Validation of cortisol and alpha-amylase as stress indicators in tinnitus

In this study, a divergence was observed between alpha-amylase and cortisol findings. The absence of a cortisol increase may be attributed to the predominance of acute, rather than chronic, stress in the experimental setting. Alternatively, it is possible that the timing of sample collection did not capture cortisol’s slower response dynamics. Future research that isolates variables potentially interfering with these outcomes could help validate these biomarkers as reliable indicators of stress impact in tinnitus (Maruyama et al., 2012).

## 6 Limitations of the study

This study has certain limitations that should be acknowledged. Although the sample size complies with the number approved by the local ethics committee and reflects the average annual clinical population of tinnitus patients at the Otorhinolaryngology Department of Hospital Virgen de la Arrixaca (Murcia, Spain), recruitment was limited to a single clinical center within a specific geographic region. Additionally, no *a priori* sample size calculation was performed, which may limit the statistical power of the findings. This may reduce the broader applicability of the results, as regional, sociocultural, or environmental factors could influence the clinical characteristics and responses of tinnitus patients. Future research should consider involving multiple hospitals or research institutions across different geographic areas to enhance external validity.

Another limitation concerns the age distribution of the sample. While mean age was comparable across groups and showed no significant difference, the overall age range was relatively narrow, and age was not included as a covariate in the statistical analyses. Since age is known to influence both auditory processing and physiological stress responses, future studies should aim to expand the age range and explicitly consider the role of age as a moderating variable.

Despite these considerations, the present study offers valuable insights into the cognitive, emotional, and physiological correlates of tinnitus and highlights the relevance of a multimodal, gender-sensitive approach to its assessment.

Furthermore, no formal correction for multiple comparisons was applied, due to the exploratory nature of the analyses and the sample size limitations. As a result, the potential inflation of Type I error should be considered when interpreting the findings. Future studies with larger samples should implement appropriate correction methods (e.g., Holm–Bonferroni) to enhance statistical robustness.

Audiometric testing was not performed for the participants in this study. In future studies in this field, participants should perform both standard tonal audiometry and speech recognition tests.

## 7 Conclusion

In conclusion, this study provides preliminary evidence of a potential impact of tinnitus on auditory perception, text comprehension, and physiological responses under varying listening conditions. The findings suggest that tinnitus may interfere with cognitive processes and modify the perception of auditory pleasantness, particularly in quiet or moderately noisy environments. However, these results should be interpreted with caution considering the study’s methodological limitations.

The overall increase in salivary amylase in response to noise exposure underscores the importance of considering acute stress with the activation of the adrenergic system as a crucial factor in auditory disorder management, including tinnitus. This supports the multidisciplinary approach advocated in the Guidelines for Management of Tinnitus (Patil et al., 2023).

The observed gender differences in perceived difficulty and the incongruity between subjective and objective measures of pleasantness underscore the necessity of incorporating gender as a key variable in tinnitus and auditory perception research. These insights may inform the development of tailored intervention strategies.

Future research should delve deeper into the underlying mechanisms of these discrepancies and further investigate gender-based variations in noise perception and response in individuals with tinnitus. Additionally, exploring interventions designed to mitigate stress and enhance noise tolerance in tinnitus patients would be highly beneficial.

The integrative approach employed in this study—combining subjective, cognitive, and physiological assessments—confirming the efficacy of our precedent studies (Cartocci et al., 2023; Inguscio et al., 2024) serves as a potential model for future investigations in audiology and tinnitus management. This multimodal framework may contribute to a more comprehensive understanding of tinnitus experiences and facilitate the creation of more effective, personalized intervention strategies.

## Data Availability

The raw data supporting the conclusions of this article will be made available by the authors, without undue reservation.
