# Supplementary material for: Listening effort and stress in tinnitus: a multidimensional approach
Source: Front Neurosci. 2025 Jul 7;19:1591622. doi: 10.3389/fnins.2025.1591622 (PMC12277253; doi:10.3389/fnins.2025.1591622)
Supplement: Supplementary file 1 [file Table_1.docx]

**PREGUNTAS DE COMPRENSIÓN – CAPÍTULO PRIMERO DE DON QUIJOTE**

1. ¿En qué región se ambienta la historia?
   a. Murcia
   b. Mancha
   c. No se quiere mencionar el nombre
   d. Comunidad de Madrid
2. ¿Qué tenía en el estante?
   a. Todas son correctas
   b. Un escudo
   c. Un rocín
   d. Una lanza
3. ¿Qué comía el narrador el sábado?
   a. Carne cruda
   b. Lentejas
   c. Huevos con jamón
   d. Pan
4. ¿Cómo proveía el hombre a su decoro durante los días de la semana?
   a. Con la mejor lana burda
   b. Traje de terciopelo
   c. Lana de baja calidad
   d. Cambiaba cada día
5. ¿Con quién vivía el hombre?
   a. Solo
   b. Con su esposa
   c. Con su padre
   d. Con una ama, su sobrina y un mozo
6. ¿Quién se ocupaba del exterior?
   a. El mozo
   b. La institutriz
   c. Un jardinero
   d. El protagonista
7. ¿Quién es el hombre de la historia?
   a. Un marinero
   b. Un zapatero
   c. Un caballero
   d. Un comerciante
8. ¿Cuántos años tiene?
   a. Sesenta
   b. Cuarenta
   c. No llegaba a los cincuenta
   d. No se dice
9. ¿De qué era amante el caballero?
   a. La pesca
   b. Caminar
   c. Levantarse temprano y la caza
   d. La lectura
10. ¿Qué tipo de libros leía cuando no tenía nada que hacer?
    a. Novelas
    b. No leía
    c. Libros de misterio
    d. Libros de caballería
11. ¿Qué le ocurrió al hombre por tanto leer?
    a. Perdió el juicio
    b. Se volvió loco
    c. Se volvió orador
    d. Nada
12. ¿Qué vendió el caballero?
    a. Parte de sus bienes
    b. Sus tierras
    c. Todos sus bienes
    d. Nada
13. ¿Qué hacía por la noche?
    a. Dormía
    b. Trabajaba
    c. Pasaba la noches leyendo
    d. Estudiaba
14. ¿Qué idea tuvo después de leer?
    a. Ser escritor
    b. Seguir trabajando
    c. Hacerse caballero errante
    d. Ninguna
15. ¿Por qué tomó esta decisión?
    a. Para cambiar de vida
    b. Por pasión
    c. Para hacerse famoso
    d. Para honrar su nombre y servir a la patria
16. ¿Qué fue lo primero que hizo?
    a. Compró un libro nuevo
    b. Limpió unas armas viejas
    c. Compró equipamiento
    d. Compró un caballo
17. ¿Cómo llamó al caballo?
    a. Ruspante
    b. Rocín
    c. Rocinante
    d. Rusprante
18. ¿Qué decidió hacer después de nombrar al caballo?
    a. Comenzó su viaje
    b. Se mudó
    c. Compró ropa nueva
    d. Cambió su nombre
19. ¿Cuántos días pasaron antes de hacerlo?
    a. Dos
    b. Cinco
    c. Ocho
    d. Diez
20. ¿Qué le faltaba en ese momento?
    a. Una dama de la que enamorarse
    b. Nada
    c. Una comida caliente
    d. Un arma nueva
21. ¿Cómo se llamaba la campesina?
    a. Ambra Lionesi
    b. Ángela Liutisi
    c. Aldonza Lorenzo
    d. No lo sabía
22. ¿De dónde era originaria la joven?
    a. Del lugar
    b. De El Toboso
    c. Castilla y León
    d. De fuera de España
23. ¿Qué nombre le dio a la doncella?
    a. Dulcinea
    b. Dulcinea del Toboso
    c. Aurora del Turoso
    d. No le dio ningún nombre
